# Bilateral striopallidal calcinosis secondary to systemic lupus erythematosus

**DOI:** 10.1016/j.radcr.2022.03.114

**Published:** 2022-04-25

**Authors:** Juan Felipe Betancur, José A. Goméz-Puerta, Juan Felipe Llano, Gustavo Adolfo Lopéz-Ochoa, Beatriz Ramirez, Oscar David Martinez

**Affiliations:** a Internal Medicine Department, Clínica Medellín QuirónSalud/Salúd SURA, Carrera 65B No. 30 - 95, Medellín, Colombia; b Rheumatology Department, Hospital Clínic Barcelona, Barcelona, Spain; c Clinical Research Department, Clínica Medellín QuirónSalud, Medellín, Colombia; d Epidemiology Department, Clínica Medellín QuirónSalud, Medellín, Colombia; e Neurology Department, Clínica Medellín QuirónSalud, Medellín, Colombia

**Keywords:** Lupus erythematosus, Systemic, Systemic lupus erythematosus, Central nervous system, Bilateral striopallidadodentate calcinosis, Basal ganglia calcification

## Abstract

Bilateral symmetric striatopallidal calcinosis with or without deposits in dentate nucleus, thalamus, and white matter is reported in patients ranging from asymptomatic, metabolic, toxic, and genetic autosomal dominant, familial or sporadic forms. Of the connective tissue diseases, it has been reported in very few cases in patients with systemic lupus erythematosus, many incorrectly labeled as Fahr syndrome without even having hypoparathyroidism. Here we describe a 30-year-old female patient with neuropsychiatric systemic lupus erythematosus manifested at diagnosis with mood disorders and anxiety, and 1-year later develops Lupus headache; Incidentally, an aneurism of the right middle cerebral artery and bilateral and symmetric calcifications of the caudate and lenticular nuclei were noted; this finding is a rarely reported manifestation of neuropsychiatric systemic lupus erythematosus.

A review of the literature based on this case was carried out in electronic databases. There are approximately 29 patients reported in the literature, with calcifications in the basal ganglia associated with systemic lupus erythematosus occurs almost exclusively in young women (96.5%) with a mean age of 33.36 years (2 months-76 years), with a race predilection for Asians (65.5%). Regarding the neuropsychiatric syndromes defined by the American College of Rheumatology, the most frequently associated are movement disorders; followed by cognitive dysfunction, seizure disorders, mood disorders, cerebrovascular disease, psychosis, and acute confusional state, transverse myelitis, and demyelinating syndrome. The mean duration time of the SLE to detection of the basal ganglia calcification is 7.62 years (3 days-31 years).

## Introduction

Central nervous system involvement in Systemic Lupus Erythematosus (SLE) or Neuropsychiatric SLE (NPSLE) comprises a variety of neurological and psychiatric presentations, with a prevalence ranging from 21% to 95%, with a widely accepted average of 40%-50%. Only one-third of the events can be attributed directly to the SLE (Primary NPSLE) or as a consequence of the complications of the disease or its treatment (Secondary NPSLE) [1]. The diagnosis is clinical, as there is no reliable method for its diagnosis.

## Case description

**Fig. 1:**
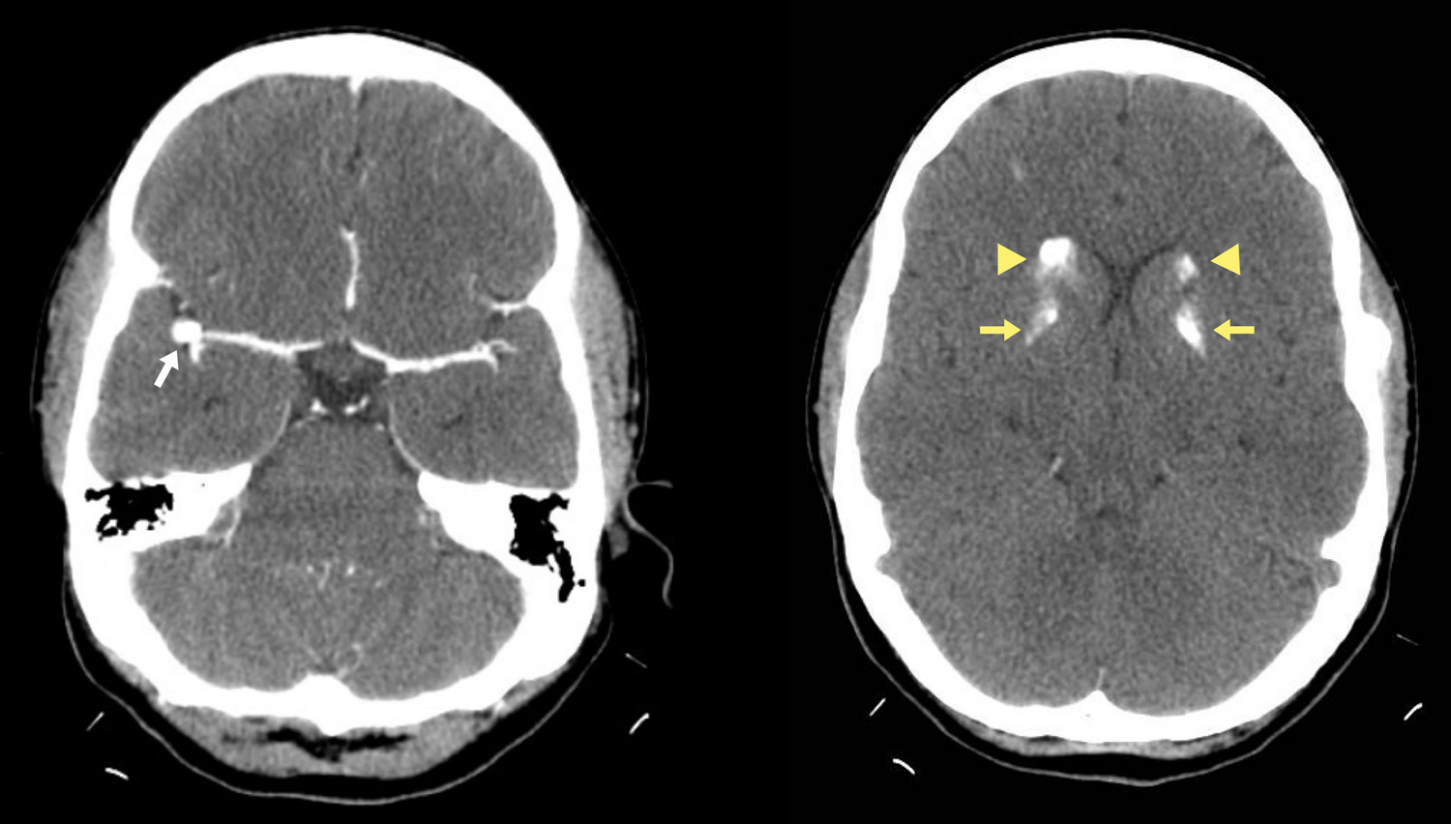
Contrast-enhanced head CT: showing an aneurism of the right middle cerebral artery at the angle of M2-M3 (Fig. 1A). Incidentally, bilateral and symmetric calcifications of the caudate and lenticular nuclei compatible with Fahr disease were noted.

**Fig. 2:**
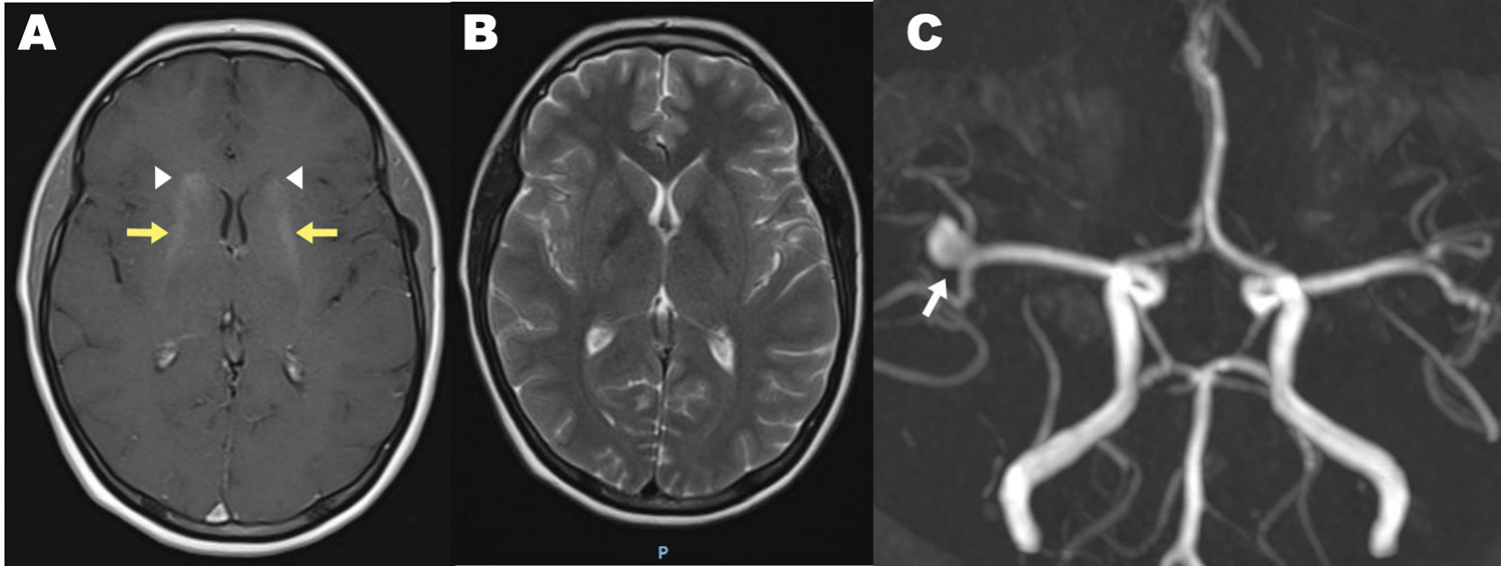
Contrast-enhanced head MRI: (A) Axial T1 weighted: bilateral symmetrical abnormal hyperintensities in the bilateral basal ganglia (white arrowheads) and corona radiata (yellow arrow). Absent in T2W images suggestive of calcifications. (B) Axial T2 weighted MRI: Absence of hyperintensities indicating lack of vasogenic or cytotoxic edema. (C) Time of Flight (TOF): Saccular aneurysm of the M2 portion of the right middle cerebral artery is observed with a longitudinal diameter of 9 mm, neck 3 mm, and dome of 7 mm.

A 30-year-old female patient with a history of Systemic Lupus Erythematosus was diagnosed 1 year ago based on the *American College of Rheumatology* (ACR) / *European League Against Rheumatism* (EULAR) 2019 criteria. Back then, she met immunologic (with positive anti-nuclear antibodies AC-1 homogeneous pattern 1:2560, ENA: Ro positive), hypocomplementemia C3:0.2 (range 90-207) C4: 14.2 (range 17.4-52.2), articular compromise criteria, she was also diagnosed with anxiety disorder and depression. In treatment with prednisolone 5 mg QD, hydroxychloroquine 200 mg QD, methotrexate 15 mg each week, and escitalopram 10 mg QD. The patient was referred to our institution for 2 months of persistent, severe, and disabling headache, holocranial in distribution, with pain Visual Analog Scale (VAS) 10/10, without aura, sonophobia, or photophobia, unresponsive to non-steroidal anti-inflammatory drugs (NSAIDs), and opioids. A contrast-enhanced head computed tomography (CT) is performed, showing an aneurism of the right middle cerebral artery at the angle of M2-M3 of 5.6 mm with a neck of 2.2 mm (Fig. 1A). Incidentally, bilateral and symmetric calcifications of the caudate and lenticular nuclei compatible with Fahr disease were noted (Fig. 1B), and Gadolinium magnetic angioresonance (angioMRI) is performed to better characterization of the lesions (Fig. 2). Despite these findings, the cause of the headache was not thought to be related to the aneurysm. She underwent surgical resection of her aneurysm, without post-operatory complications. The headache persisted in the early postoperative period and did not subside with opioids. Post-surgical meningitis, intracranial hypertension, and hemorrhagic and thrombotic complications were ruled out. The patient has no meningeal, parkinsonism, or Parkinson plus symptoms; only a mild cognitive impairment with compromise in attention, psychomotor slowdown, and worsening of her anxiety and depression were noted. The headache was presumed due to SLE. Relevant laboratory findings were: anti-DNA-ds:1:10 (<1:10) C3: <11 mg/dL (83-193) C4:17 mg/dL (15-57) hemogram without anemia, leukopenia or thrombocytopenia, the antiphospholipid syndrome profile was negative: B2-glycoprotein I IgM:3.2 IgG:5.29 anti-cardiolipins: IgM: 6.4 IgG:8.9 Lupus Anticoagulant (DRVVVT): patient:31.9 seconds control 29.3 seconds screen: 1.03 (0.8-1.2) Cr 0.57 mg/dL with normal uroanalysis, the calculated Systemic Lupus Erythematosus Activity Index (SLEDAI) 2K was:10 points. Causes of basal ganglia calcification including hypoparathyroidism were ruled out: Calcium:9.18 mg/dL phosphorus:3.9 mg/dL Parathormone 53.2 pg/mL (10.8-79.4 pg/mL) VDRL, Hepatitis B virus surface antigen and hepatitis C virus: were negative, Epstein-Barr Virus VCA antibodies: IgM: 12.4 U/mL (<20) Toxoplasma gondii antibodies: IgM: 0.17 S/CO (<0.83 S/CO) Cytomegalovirus IgM 0.57 COI (0-0.84). It was concluded that we were in the context of a lupus headache, treated with pulses of methylprednisolone 500 mg every day for 3 days; the pain improved on the second day of treatment, with cessation of this on the fourth day of treatment.

## Discussion

Evaluating the brain structure through neuroimaging is one of the mandatory procedures in a patient with NPSLE since they help locate intracranial abnormalities, the compromise of the white or gray matter, and assess its evolution over time. Brain tomography can be abnormal in 29%-59% of NSPSLE patients; the most common finding is regional or global brain atrophy and white and gray matter changes [2]. It is also helpful to determine the presence of extensive infarcts, intracerebral bleeding, and diffuse cerebral edema and exclude confounders such as brain abscesses, meningitis, mycotic aneurysm, or masses [2]. Nevertheless, due to its sensitivity (40%-75%), Magnetic Resonance Imaging (MRI) is the gold standard in the evaluation of NPSLE, where advanced MRI techniques such as diffusion-tensor, magnetization transfer, and volumetric studies, which give microstructural and functional information, with evidence of subtle brain changes even in subclinical levels [1].

Cerebral calcifications in patients with neuropsychiatric involvement due to SLE have a predilection for the basal ganglia and less frequently in the cerebral white matter and the cerebellum [3]. When they present, they are always bilateral symmetric, always affecting the globus pallidus, followed by the cerebellum, putamen, the semioval center, the head of the caudate, and the thalamus. Radiologically, these calcifications are presented in proportions similar to those observed in hypoparathyroid patients, which probably leads to these patients being erroneously labeled with Fahr's disease/syndrome (a misnomer) [4], [5], [6], [7]. Only 1 reported case of intracerebral calcifications in the literature can be genuinely attributed to Fahr's syndrome [8]. The most appropriate name for this condition is bilateral striopallidodentate calcinosis secondary to SLE.

The first case reported in the literature of ganglio-basal calcifications in SLE was evidenced in 1979 by Gonzalez-Scarano F et al. in a case series of brain tomography in 29 patients with a clinical compromise of the central nervous system [9].

There are approximately 30 patients reported in the literature, including ours with calcifications in the basal ganglia associated with SLE [3,4,[14], [15], [16], [17], [18], [19], [20], [21], [22], [23],^6^,^24^,^25^,[7], [8], [9], [10], [11], [12], [13]], to date; it occurs almost exclusively in young women (96.5%) with a mean age of 33.36 years (2 months-76 years), with a race predilection for Asians (65.5%), followed by Caucasian (24.1%) Afroamerican (6.9%) and Mestizo (3.5%). Regarding the neuropsychiatric syndromes defined by the ACR, the most frequently associated are movement disorders in 27.5% of the patients; followed by cognitive dysfunction and seizure disorders in 24.1%, mood disorders in 20.6%, cerebrovascular disease in 17.2%, psychosis 13,8% acute confusional state 10.2%, and transverse myelitis and demyelinating syndrome in 3.5%. There is usually no relationship between the presence and degree of calcification and the age of the patients, type, and duration of the neuropsychiatric syndrome [15]. The mean duration time of the SLE to detection of the basal ganglia calcification is 7.62 years (3 days-31 years)

At the laboratory level, the patients usually have high antinuclear antibodies (ANA) and anti-DNA titers, hypocomplementemia, and P-ribosomal in 17.2% of the patients with a low frequency of antiphospholipid antibodies (3.2%). One interesting finding is that 13.8% of the patients had Neonatal Lupus with 1 positive for P-ribosomal (although it was only measured in 1 patient).

Its pathogenesis to date is unknown, but primary immunological vascular damage that triggers microinfarctions with posterior dystrophic calcifications is presumed to be secondary. What is observed in these patients may be the final spectrum or consequence of a neuropsychiatric compromise that occurs early in the disease in which autoantibodies directed against antigens located in the basal ganglia damage the basal ganglia cells (the core of the lesion), followed by changes in vascular lesions in the basal ganglia (peripheral vasogenic edema) [26]. These lesions are reversible with early immunosuppressive treatment. Not all patients present extra-pyramidalism since acute inflammation does not necessarily lead to dysfunction of the basal ganglia, and this would only appear in some patients in whom there would be anti-basal ganglia antibodies that affect neuronal function [26].

Regarding treatment, little has been described, and they have been managed conventionally with steroids (prednisone 1 mg/Kg). However, a case report of an early compromise of the basal ganglia manifested as basal ganglia involvement manifested as hyperintensities in T2W / FLAIR (vasogenic and cytotoxic edema) were reversible with pulses of steroids and cyclophosphamide. If high-intensity lesions are present in T1W, these may represent calcifications and a more advanced stage of their involvement in which aggressive immunosuppression may not be as successful [26]. In patients with a high index of suspicion with apparently normal MRI, advanced techniques MRI as morphometric methods, diffusion-tensor, magnetization transfer imaging, spectroscopy, perfusion MRI or quantitative MRI susceptibility mapping are recommended [27,28].

## Conclusions

Neuropsychiatric SLE compromise almost half of the patients as primary or secondary NSPSLE. The diagnosis is eminently clinical, but paradoxically, subclinical manifestations can be detected prematurely in neuroimaging, mainly MRI. With the advent of new MRI techniques, this subclinical compromise is detected earlier and allows these patients to be treated earlier to avoid brain damage, improving the quality of life in patients with this devastating multisystemic disease.

## Patients consent

Informed written consent was obtained from the patient for publication of the Case Report and all imaging studies. Consent form on record.

## Compliance with ethical standards

This article is not funded.
